# Zinc transporter ZIP12 maintains zinc homeostasis and protects spermatogonia from oxidative stress during spermatogenesis

**DOI:** 10.1186/s12958-022-00893-7

**Published:** 2022-01-22

**Authors:** Xinye Zhu, Chengxuan Yu, Wangshu Wu, Lei Shi, Chenyi Jiang, Li Wang, Zhide Ding, Yue Liu

**Affiliations:** 1 Department of Clinical Medicine, Shanghai Jiao Tong University School of Medicine, Shanghai, 200025 China; 2 Department of Histology, Embryology, Genetics and Developmental Biology, Shanghai Key Laboratory for Reproductive Medicine, Shanghai Jiao Tong University School of Medicine, Shanghai, 200025 China

**Keywords:** ZIP12, Zinc transport, Spermatogonia, Spermatogenesis, Oxidative stress, Antioxidant, Male infertility

## Abstract

**Background:**

Overwhelming evidences suggest oxidative stress is a major cause of sperm dysfunction and male infertility. Zinc is an important non-enzymatic antioxidant with a wide range of biological functions and plays a significant role in preserving male fertility. Notably, zinc trafficking through the cellular and intracellular membrane is mediated by specific families of zinc transporters, i.e., SLC39s/ZIPs and SLC30s/ZnTs. However, their expression and function were rarely evaluated in the male germ cells. The aim of this study is to determine and characterize the crucial zinc transporter responsible for the maintenance of spermatogenesis.

**Methods:**

The expression patterns of all 14 ZIP members were characterized in the mouse testis. qRT-PCR, immunoblot and immunohistochemistry analyses evaluated the ZIP12 gene and protein expression levels. The role of ZIP12 expression was evaluated in suppressing the sperm quality induced by exposure to an oxidative stress in a spermatogonia C18–4 cell line. Zip12 RNAi transfection was performed to determine if its downregulation altered cell viability and apoptosis in this cell line. An obese mouse model fed a high-fat-diet was employed to determine if there is a correlation between changes in the ZIP12 expression level and sperm quality.

**Results:**

The ZIP12 mRNA and protein expression levels were higher than those of other ZIP family members in both the mouse testis and other tissues. Importantly, the ZIP12 expression levels were very significantly higher in both mice and human spermatogonia and spermatozoa. Moreover, the testicular ZIP12 expression levels significantly decreased in obese mice, which was associated with reduced sperm zinc content, excessive sperm ROS generation, poor sperm quality and male subfertility. Similarly, exposure to an oxidative stress induced significant declines in the ZIP12 expression level in C18–4 cells. Knockdown of ZIP12 expression mediated by transfection of a ZIP12 siRNA reduced both the zinc content and viability whereas apoptotic activity increased in the C18–4 cell line.

**Conclusions:**

The testicular zinc transporter ZIP12 expression levels especially in spermatogonia and spermatozoa are higher than in other tissues. ZIP12 may play a key role in maintaining intracellular zinc content at levels that reduce the inhibitory effects of rises in oxidative stress on spermatogonia and spermatozoa viability during spermatogenesis which help counteract declines in male fertility.

**Supplementary Information:**

The online version contains supplementary material available at 10.1186/s12958-022-00893-7.

## Background

Infertility in humans is defined as a failure to result in pregnancy after 12 months of unprotected sexual intercourse. This dysfunction is reported to affect declines in approximately 15% of the couples according to a recent epidemiological study [1, 2]. In these cases, up to 50% of all infertility is associated with male factors, mainly caused by declines in sperm concentration and motility that accompany defects in morphology [3]. As an etiological factor, excessive reactive oxygen species (ROS) generation imposes an oxidative stress that contributes to between 30 and 80% of cases of male infertility [4, 5]. ROS is a group of oxygenated free radicals, including superoxide anions (•O^2−^), hydrogen peroxide (H_2_O_2_), proxyl (•OOR), and hydroxyl (•OH) [6]. Excessive ROS generation that overwhelms endogenous antioxidant defenses can lead to nuclear DNA damage and mitochondrial dysfunction, lipid peroxidation, shortening of telomere length, and epigenetic changes in target cells [7, 8].

Spermatogenesis commonly refers to the process of creating mature male viable gametes, which is a continuous and sophisticated cell differentiation process that involves a wide repertoire of spermatogenic cells, including spermatogonia, spermatocytes, spermatids, and spermatozoa [9]. Overwhelming evidence suggests that excessive ROS generation is a major cause of impaired spermatogenesis and sperm dysfunction, and acts as a detrimental factor in the etiology of male infertility owing to impairment of both the structural and functional integrity of spermatozoa [1, 10]. Since sperm cells have most of their cytoplasm extruded and their transcription is generally repressed, they have neither the cytoplasmic storage capacity nor the transcriptional ability to blunt excessive rises in ROS generation induced by environmental challenges. Therefore, excessive ROS generation must be continuously inactivated by antioxidants in the genital tract, such as zinc [11].

Zinc is a member of a family of non-enzymatic antioxidants and it exerts significant roles through multiple mechanisms in the antioxidant network. They include regulation of oxidant production, maintaining the activity of Cu/Zn-superoxide dismutase (SOD), modulation of zinc-regulated transcription factors, increasing metallothionein expression, regulation of GSH metabolism, modulation of protein kinases and phosphatases and regulation of redox signaling [5, 12, 13]. Notably, the zinc content is relatively high in the male reproductive system and exerts essential roles in maintaining spermatogenesis and protecting the testis against oxidative stress. For instance, zinc is a co-factor of metalloenzymes for most enzymatic reactions [1, 12], which are involved in DNA replication and packaging, DNA transcription, steroid receptor expression, and protein synthesis [13–16]. Meanwhile, intracellular zinc content in the developing spermatogenetic cells is upward during spermatogenesis and plays the main role in the adjustment of the spermatogonia proliferation, spermatocytes meiosis, and preservation of the epithelial integrity of the seminiferous epithelium [10–12, 17–19]. Moreover, zinc can mitigate testis injury induced by stressors such as heavy metals, fluoride, and heat [12]. On the other hand, zinc deficiency is associated with abnormal testicular endocrine function [14, 20], impaired spermatogenesis [1, 21], and decreases in sperm concentration of the ejaculate [14]. In the clinics, including a dietary zinc supplement is reported to beneficial in therapeutic management of male infertility although the relevant data were sometimes contradictory [5, 22–25].

Notably, to sustain homeostasis of zinc, two families of proteins are responsible for the transport of Zn^2+^ across the cellular and intracellular membranes in opposite directions [26]. The zinc transporter proteins (ZnT; encoded by SLC30a gene family) transport Zn^2+^ out of the cytosol, whereas the Zrt-, Irt-like proteins (ZIP; encoded by SLC39a gene family) transport Zn^2+^ into the cytosol from the intracellular compartments or the extracellular fluid [27]. These two types of zinc transporters are conserved in mammals [28]. Human genome sequencing has identified 10 members of ZnT family (referred to as ZnT1–10) and 14 members of ZIP family (referred to as ZIP1–14) [15]. It was reported that decreased zinc intake is associated with spermatogenesis deficiency, but this conclusion was arrived at without any data describing circulating and testicular zinc levels [15]. Nevertheless, dysfunction of zinc transporters was attributed to underlie declines in spermatic vitality and genesis.

Herein, we categorize the relative contributions by members of the ZIP transporter family to the maintenance of sperm homeostasis and vitality in the male reproductive system. The results indicate that ZIP12 activity has a prevailing essential role in blunting increases in ROS generation that otherwise would suppress both spermatogenesis and fertility.

## Methods

### Animals and obese model

The male 3-week-old C57BL/6 mice were purchased from the Shanghai Laboratory Animal Center and acclimated in the animal facility for at least 1 week before experimentation. Male mice were randomly divided into two groups: the control diet (CD) group and the high-fat diet (HFD) group. The control diet contained 50% carbohydrate, 22% crude protein, 4% crude fat, 5% cellulose, 8% minerals, 1% vitamins, 10% water, whereas the high-fat diet contained 20% lard, 25% starch, 15% dextrose, 5% sucrose, 8% casein, 2% cholesterol, 1% cholate, 5% cellulose, 5% soybean oil, 5% minerals, 1% vitamins, 8% water. All of the mice were weighed every week. Both groups had ad libitum food and water access and were maintained on a 12 h light and 12 h darkness cycle. The mice fed with CD or HFD for 10 weeks were employed for animal experiments [29]. Animal experiments were conducted according to the International Guiding Principles for Biomedical Research Involving Animals, as promulgated by the Society for the Study of Reproduction. This research program was approved by the ethics committee of Shanghai Jiao Tong University School of Medicine (No. A2019–029).

### Gene expression analysis

Mice hearts, livers, spleens, lungs, kidneys, brains, small intestines, testes, C18–4 cells, and GC-1 cells were homogenized in the TRIzol reagent (Invitrogen, US). cDNA was reverse transcribed from 1 μg RNA using PrimeScript RT Master Mix (TaKaRa, Japan). SYBR green-based quantitative reverse transcription-polymerase chain reaction (RT-qPCR) was used to measure the expression of ZIPs in mouse testes and the ZIP12 expression levels were ranked in their different organs. Primers used for PCR are listed in Supplementary Table 1. PCR conditions were set as follows, 95 °C for 5 min, followed by 40 cycles at 95 °C for 15 s, and 60 °C for 43 s. Each PCR was run at least in triplicate. Finally, the data were analyzed by using the 2^−ΔΔCT^ method to measure the relative gene expression levels.

### Western blot analysis

Protein from C18–4 cell lysates or mouse tissues was resolved by performing 10% SDS-polyacrylamide gel electrophoresis (SDS-PAGE). Then polyvinylidene difluoride (PVDF) membranes (Millipore, Germany) were used to transfer the protein. The membranes were blocked by using 5% bovine serum albumin (BSA) for 1 h, then incubated at 4 °C overnight with the primary antibodies against either ZIP12 (Abcam, 1:5000) or β-actin (Abcam, 1:5000), followed by incubation with secondary antibody conjugated to HRP (Jackson, West Grove, PA, USA, 1:10000 dilution). Then enhanced chemiluminescence (Millipore, Germany) was used to generate the signals detected by a Luminescent Image Analyzer (Image Quant LAS 4000, GE imagination at work, USA) according to the manufacturer’s protocol. Western blot were repeated at least three times to confirm the reproducibility of a result [29, 30].

### Immunohistochemistry (IHC) analysis

IHC was performed as described previously [31]. Tissues fixed in Bouin’s solution were embedded in paraffin. Then, specimens were sliced into 5 μm thick sections and mounted on glass slides, followed by deparaffinization and rehydration, and subsequently followed by antigen retrieval through boiling the tissue for 15 min in 10 mM citrate buffer, pH 6.0. The Histostain LAB-SA Detection kits (Invitrogen, MD, USA) were applied according to the manufacturer’s instructions. Primary antibody exposure against ZIP12 (1:100 dilution) and the normal rabbit IgG (control) was performed overnight at 4 °C. The sections were stained using DAB and nuclei were counterstained with hematoxylin. Digital images were captured under a microscope (Olympus BX53).

### Immunofluorescence (IF) analysis

For IF staining, sperm smears or testicular sections were prepared on slides and C18–4 cells were cultured on glass-bottom dishes (Cellvis, Mountain View, CA, USA). Then, slides or dishes were fixed with 4% paraformaldehyde for 20 min at 4 °C. Nonspecific binding sites were blocked with 10% BSA/PBS for 60 min at room temperature, followed by 0.1% TritonX-100 permeable treatment for 10 min. Sections were incubated with the ZIP12 antibodies (Abcam, 1:200 dilution), and/or PLZF antibodies (Santa Cruz Biotechnology, 1:200 dilution, USA) overnight at 4 °C, respectively. Then, fluorescence-labeled secondary antibodies (donkey anti-rabbit Alexa Fluor 488 or donkey anti-mouse Alexa Fluor 555, 1:200 dilution; Jackson ImmunoResearch) were used. Nuclei were counterstained with DAPI (Sigma-Aldrich). The fluorescence signals were detected under a laser scanning confocal microscope (Carl Zeiss LSM-510, Germany) equipped with an argon laser (488 nm), a He/Ne laser (543 nm), an EC Plan-NEOFLUAR 63×/1.25 objective, and an LD LCI Plan-APOCHROMAT 25×/0.8 objective (Zeiss). Digital images were taken and processed using Aim software (Zeiss Systems) [30].

### Assessment of sperm parameters

Preparation and analysis of mouse sperm were performed as described previously [32].The cauda epididymis was dissected and then placed in pre-warmed (37 °C) Tyrode’s Buffer (Sigma-Aldrich, USA) to allow dispersion of sperm. After incubation for 15 min, supernatant up-streamed sperm were segregated and sperm motility was analyzed by computer-assisted sperm analysis (CASA) (Hamilton Thorne, USA).

For teratozoospermia analysis, the sperm suspension was initially smeared on a glass slide. After dryness, the slide was fixed and stained by the method of Diff-Quick (BRED Life Science Technology Inc., Shanghai, China) according to the manufacturer’s protocol. Finally, the slide was viewed under a microscope (Nikon, ECLIPSE E600, Japan). Sperm samples obtained from 10 HFD-fed mice and 10 normal diet-fed mice respectively were detected and at least 200 spermatozoa were included in every sample.

### Intracellular zinc analysis

For detection of zinc in sperm, up-streamed sperm suspension (1 × 10^5^ per mL) was incubated with the zinc probe, ZnAF-1 (final concentration at 1 μg/mL, Abcam) or Zinquin ethyl ester (final concentration at 25 μM, Dojindo) for 30 min at 37 °C. After washing with PBS, the fluorescence signals were detected using flow cytometry (Becton Dickinson, Beckman Coulter). Fluorescence of ZnAF-1 was excited at 485 nm and detected at 525 nm, and fluorescence of Zinquin ethyl ester was excited at 368 nm and detected at 490 nm. The CellQuest software analyzed the emission originated from at least 30,000 events (Beckman Coulter), and three repeats were performed of each sperm sample. Meanwhile, the distribution of zinc staining in sperm was viewed under fluorescence microscopy (Olympus BX53, Tokyo, Japan).

### Cell culture and RNAi experiment

The immortalized mouse spermatogonial stem cells, C18–4 cells were cultured with DMEM/F12 medium containing 10% FBS in six-well plates. The cells were divided into three groups, i.e., a blank group without any treatment, ZIP12-shRNA group transfected with mouse SLC39A12 shRNA plasmids containing an shRNA expression cassette and a green fluorescent protein (GFP) marker (obtained from Sangon Biotech, Shanghai, China), and control group transfected with control plasmids. The effective ZIP12-specific and control shRNA sequences are as follows: *ZIP12*, 5′-ctcaggttcttggtttacataagcaggaa-3′; control, 5′-gcactaccagagctaactcagatagtact-3′ [33]. Efficacy of zinc transporter ZIP12 expression knockdown was confirmed by Western blot analysis.

### Determination of intracellular ROS

The levels of intracellular ROS were evaluated using the peroxide-sensitive fluorescent probe 2′7’-dichlorofluorescein diacetate (DCFH-DA) (Dojindo, Kumamoto, Japan). The sperm were exposed to a serum-free medium containing 10 μM DCFH-DA in the dark for 30 min and then washed thrice with cold PBS. Flow cytometry was used to measure the fluorescence intensity (FACS Calibur, Becton-Dickinson, Sunnyvale, CA).

### Caspase-3 activity assay

Caspase-3 activity was measured using the Caspase-3 Activity Assay Kit (Dojindo) according to the manufacturer’s protocol. In brief, cell suspension (blank group, control group, and ZIP12-shRNA group) was inoculated in 6-well plates. After being cultured for 24 h, the cells were treated with 100 μM H_2_O_2_ for 24 h and then collected for Caspase-3 activity assay. Collected cells were lysed in the lysis buffer on ice and then lysis buffer was added to dilute cell lysate supernatant to 1 mg/ml. Then, substrate and assay buffer was added to the cell lysate supernatant and the mixture was incubated at 37 °C for 2 h. Finally, Caspase-3 activity was determined based on the relevant optical absorbance detected at 405 nm with a Multiskan GO Spectrophotometer (Thermo Fisher Scientific, Vantaa, Finland).

### Cell viability analysis

Cell suspension (100 μL per well) was inoculated in 96-well plates and cultured at 37 °C, 5% CO_2_ for 24 h. The cells from the blank group, control group, or Zip12-shRNA group were directly received 10 μL of CCK-8 solution and then incubated for 2 h. The relevant optical absorbance was detected at 450 nm with a Multiskan GO Spectrophotometer.

### Statistical analysis

All data were analyzed using SPSS software (SPSS Statistic 24, Chicago, IL, USA), and the data are reported as mean ± SEM. Group comparisons were made using Student’s *t*-test where appropriate. One-way analysis of variance (ANOVA) test was used assuming a two-tail hypothesis with *P* < 0.05. Spearman correlation analysis assessed the correlations between mean fluorescence intensity and sperm motility or progressive motility. *P* values of < 0.05 were considered statistically significant [34].

## Results

### ZIP12 is highly expressed in mouse testis

**Fig. 1:**
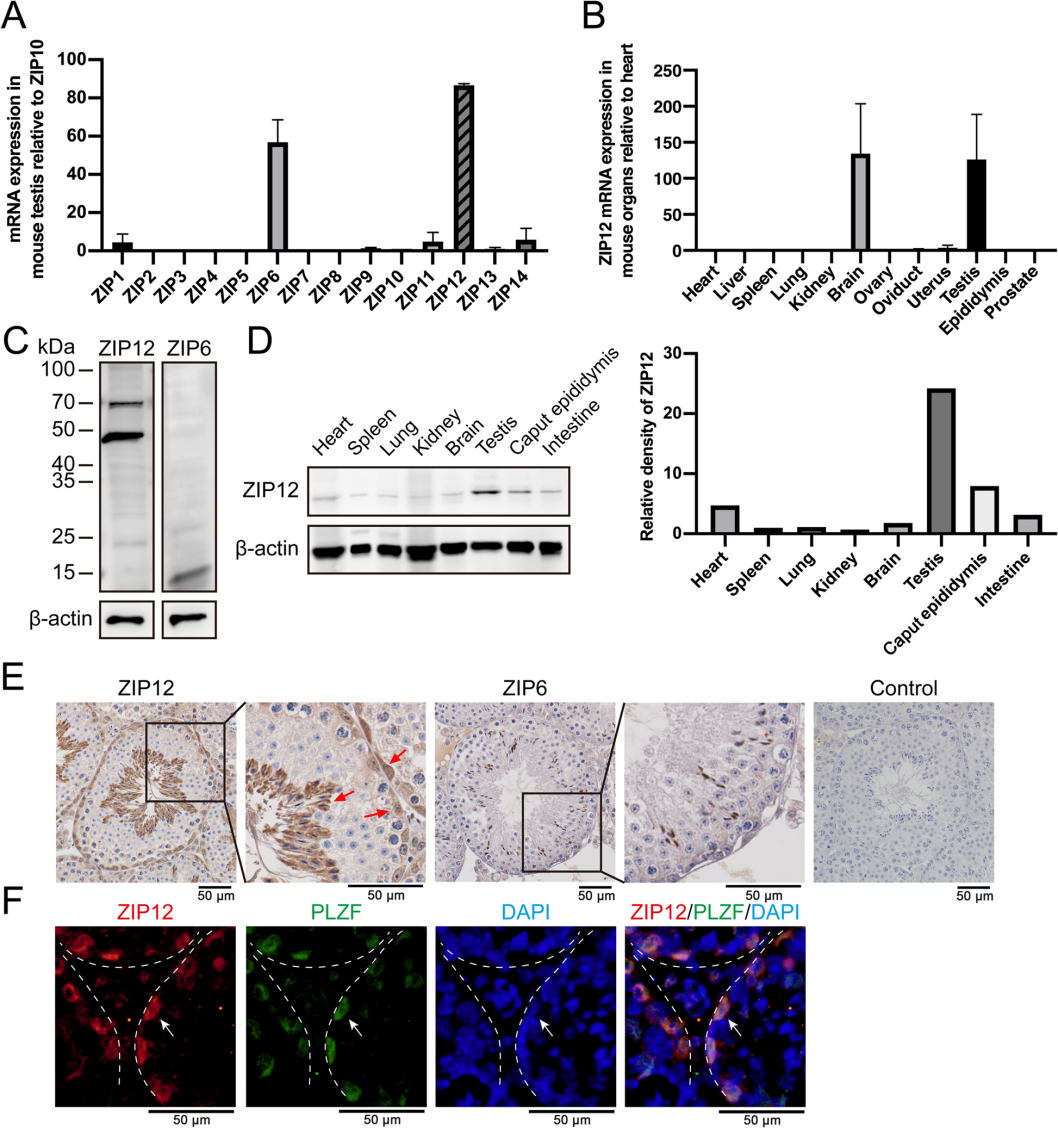
Gene and protein expression levels of ZIP12 in mouse testis and other tissues. **A** Relative expression of ZIP mRNAs detected in mouse testis by RT-qPCR. Data are presented as means±SE from three independent experiments. **B** Relative expression of ZIP12 mRNA detected in various mouse tissues by RT-qPCR. Data are presented as means±SE from three independent experiments. **C** Western blot detection of ZIP12 protein and ZIP6 protein in mouse testis respectively. The presence of protein bands of ZIP12 at 73 kDa and 46 kDa are confirmatory of ZIP12 expression. **D** Western blot detection of ZIP12 protein in the different tissues. The relative levels of ZIP12 protein were normalized to β-actin. **E** Immunohistochemical analyses of ZIP12 and ZIP6 expressions in the testis. Red arrows indicated spermatids and spermatogonia positive for ZIP12 staining. Control represents normal rabbit IgG staining. Scale bar = 50 μm. **F** Immunofluorescent staining of ZIP12 (red) in testis. Spermatogonia (white arrows) were stained with PLZF (green). Cell nuclei were stained with DAPI (blue). Scale bar = 50 μm

Since there are 14 members of the ZIP family that could transport zinc into the cytoplasm, RT-qPCR was performed to evaluate their expression levels in mice testes. The results showed that the mRNA levels of ZIP6 and ZIP12 were at a relatively high expression (Fig. 1A). Moreover, immunoblotting analysis and immunohistochemistry (IHC) staining showed that ZIP12 was abundantly expressed, whereas the expression of ZIP6 was barely visible in the testis (Fig. 1C, E). It should be noted that Western blotting detected the predicted 73 kDa and 46 kDa protein bands of ZIP12, while the 46 kDa band had a much higher optical density than the 73 kDa band.

To determine if this more prominent ZIP12 expression has possible functional significance, RT-qPCR and Western blotting were used to compare its gene and protein expression levels in different mouse tissues. The results showed that in 12 different mouse tissues including heart, liver, spleen, lung, brain, ovary, uterus, testis, epididymis, and prostate ZIP12 mRNA was profusely expressed in testis and brain Fig. 1B). This expression profile was concordant with that in Mouse ENCODE transcriptome data published in the National Center for Biotechnology Information (https://www.ncbi.nlm.nih.gov/gene/277468). Although there was a lack of correspondence between the relative gene and protein expression levels of some of these ZIP transport family members, its gene and protein expression levels were in any case relatively high in mouse testis (Fig. 1D).

Notably, IHC analysis showed that ZIP12 expression was especially localized in the spermatogonia and spermatids within the seminiferous tubules (Fig. 1E). Furthermore, the co-localization of ZIP12 IF staining with the spermatogonia marker PLZF confirmed its expression as being delimited to the spermatogonia throughout the seminiferous epithelium (Fig. 1F). Because all kinds of spermatogenic cells throughout the seminiferous epithelium are derived from spermatogonia, the high level of expression of ZIP12 in spermatogonia suggested that this isoform has a potential role in protecting spermatogonia from oxidative damage during spermatogenesis. This capability is a result of ZIP12 mediating adequate levels of intracellular Zn^2+^ uptake to reduce both ROS accumulation and losses in tissue function.

### ZIP12 localization is consistent with zinc distribution in mouse spermatozoon

**Fig. 2:**
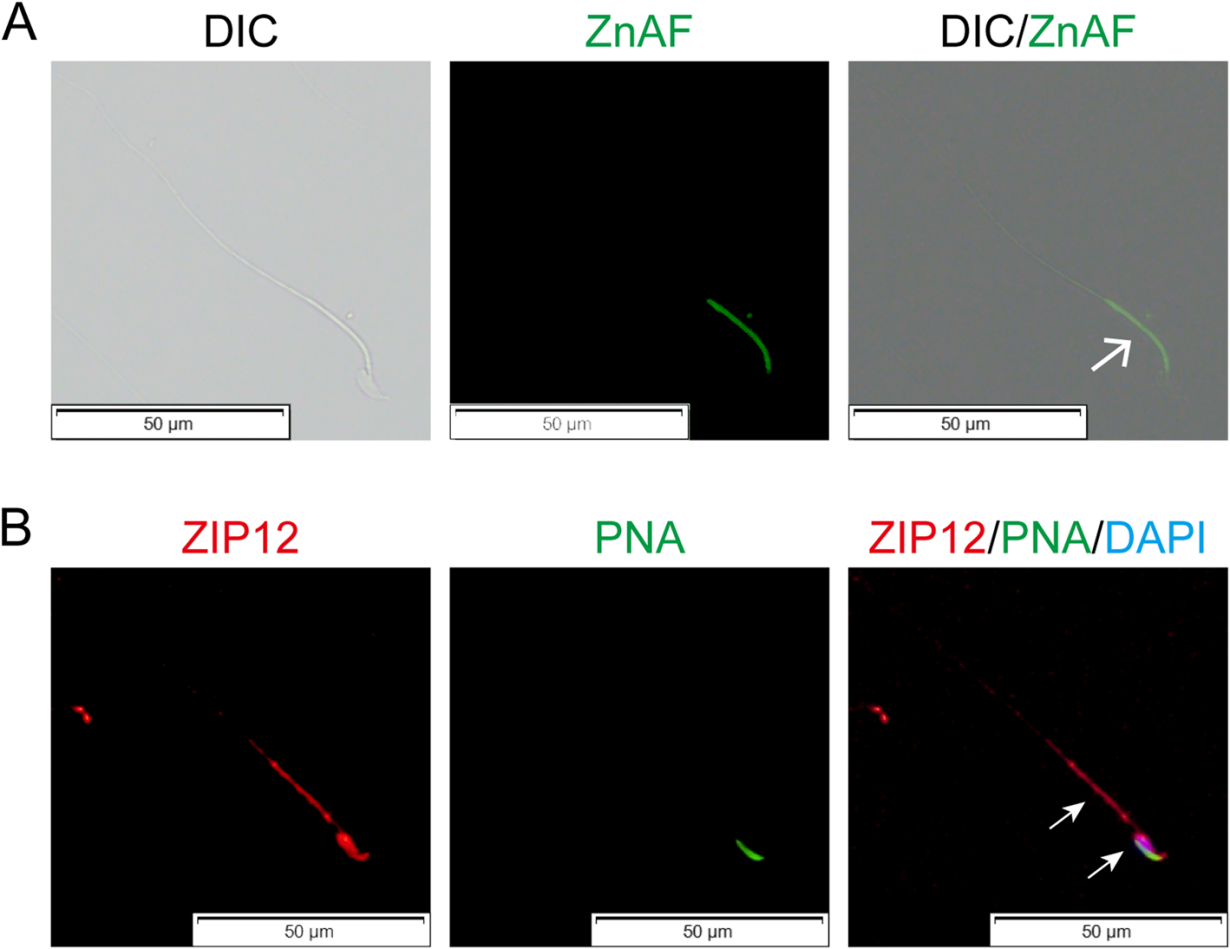
ZIP12 localization and zinc distribution in mouse spermatozoa. **A** ZnAF-1 (green) immunofluorescent staining of zinc in fresh spermatozoa. White arrow indicates zinc staining in the midpiece of the mouse sperm tail. Differential interference contrast (DIC) images show the sperm shape. Scale bar = 50 μm. **B** Immunofluorescent staining of ZIP12 (red) in mouse sperm. Acrosomes were stained with PNA (green). Cell nuclei were stained with DAPI (blue). White arrows indicated ZIP12 staining in the postacrosomal region of the sperm head and the midpiece of the sperm tail. Scale bar = 50 μm

Since sufficient levels of zinc content in spermatozoa are crucial for maintaining sperm function, we determined if there is a correspondence between ZIP12 localization and zinc distribution in spermatozoa. As shown in Fig. 2A, the distribution of zinc that was identified based on ZnAF-1 staining intensity which is a zinc specific fluorescence probe. It was primarily clustered in the midpiece of the fresh mouse spermatozoa tail. Meanwhile, the ZIP12 immunofluorescent staining pattern showed that its localization was prominent in the postacrosomal region of the sperm head and the midpiece of its tail (Fig. 2B). Their overlapping distribution profiles is supportive of a role for ZIP12 in mediating intracellular transmembrane zinc transport, which is necessary for maintaining zinc content high enough for protecting mouse spermatozoa from exposure to excessively high ROS levels.

### ZIP12 is expressed in spermatogonia and spermatozoa in humans

**Fig. 3:**
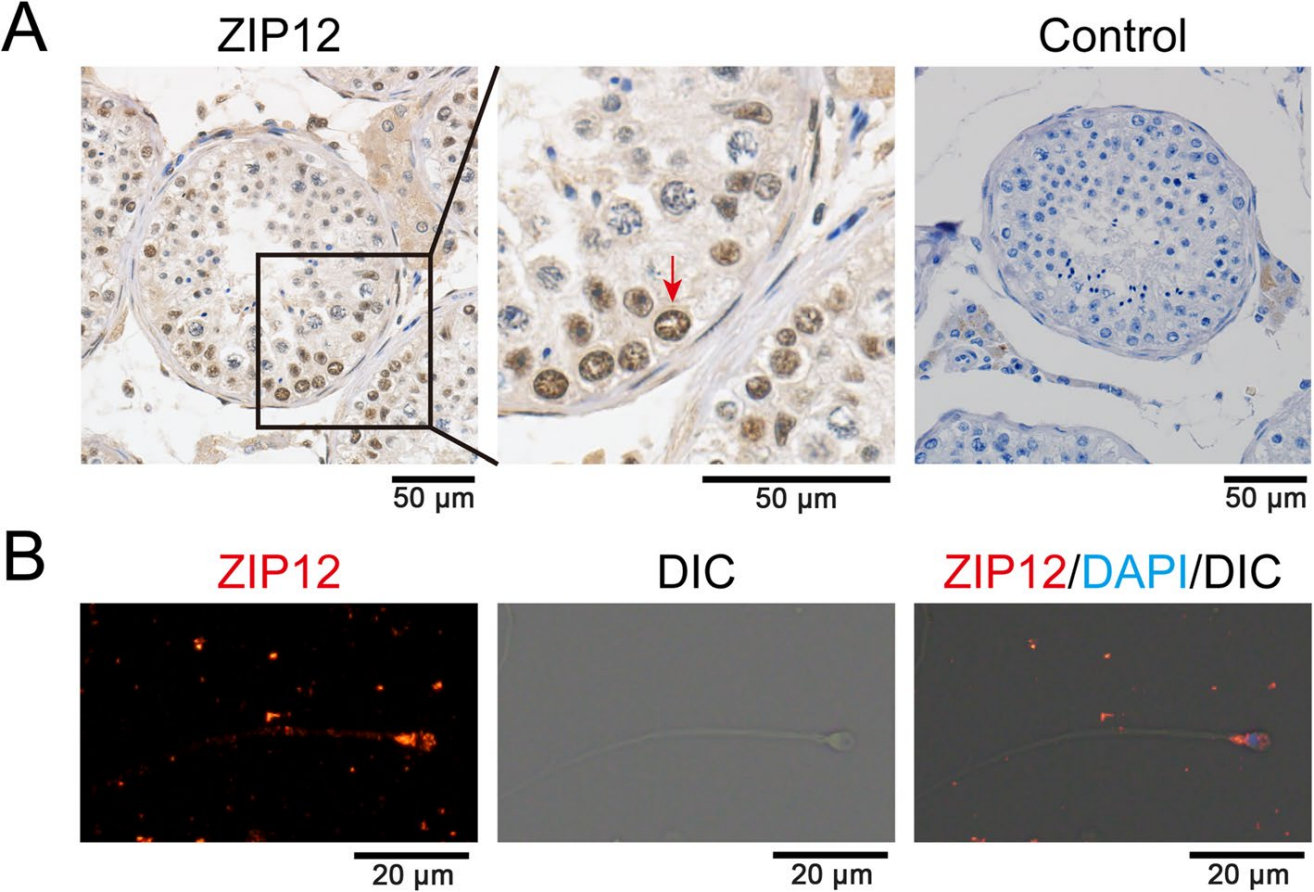
ZIP12 localization in testis and spermatozoa from human specimens. **A** Immunohistochemical analysis of ZIP12 expression in human testis. The red arrow indicated the spermatogonia positive for ZIP12 staining. Control represents normal rabbit IgG staining. Scale bar = 50 μm. **B** Immunofluorescent staining of ZIP12 (red) in human spermatozoa, indicating its distribution in the head and the midpiece of tail. Cell nuclei were stained with DAPI (blue). DIC images show the sperm shape. Scale bar = 20 μm

Slc39a12/ZIP12 expression is highly conserved across mammalian species [28]. To confirm if our characterization of the ZIP12 expression profile is relevant to that in humans, we examined its expression pattern in human testis and spermatozoa. IHC analysis was performed using testis sections from patients who received castration therapy for prostate cancer. The result showed that ZIP12 was highly localized in the spermatogonia within the seminiferous tubules (Fig. 3A). Meanwhile, the ZIP12 derived immunofluorescence was localized in the sperm head and the midpiece of the sperm tail (Fig. 3B). Therefore, these expression patterns were coincident with one another in mice and human, which suggested a similar role for ZIP12 in both mouse and human testes and spermatozoa during the spermatogenesis.

### Obesity causes downregulation of ZIP12 expression

**Fig. 4:**
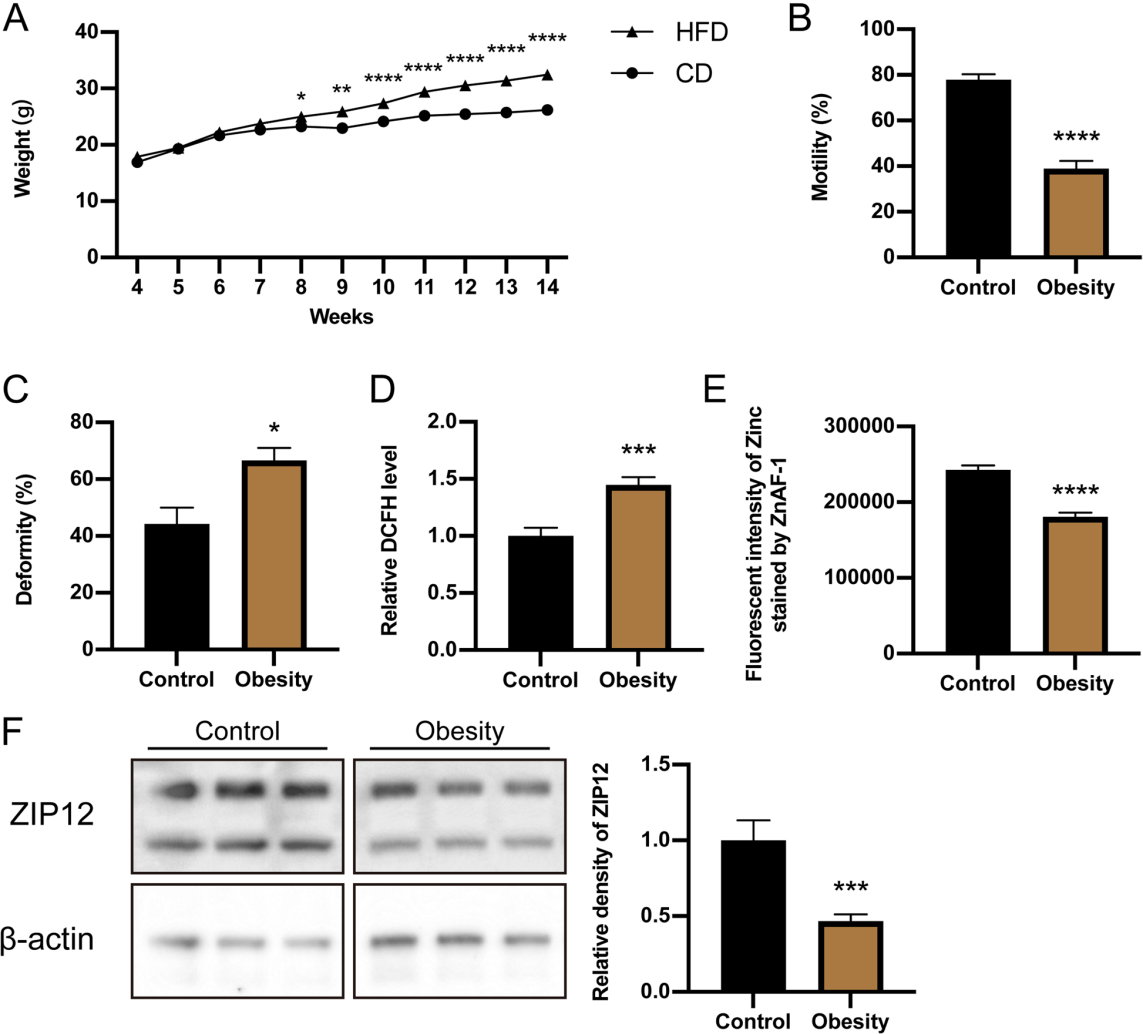
Comparison of sperm parameters, ROS level, and ZIP12 expression in obese mice. **A** Comparison of time-dependent increases in body weight between CD (*n* = 10) and HFD groups (*n* = 10). **B** Sperm motility in mice fed either CD or HFD analyzed by CASA (*n* = 10). **C** Sperm morphology analysis of CD-fed mice and HFD-fed mice using Diff-Quick method (*n* = 10). **D** Comparison of sperm ROS level in CD and HFD groups (*n* = 7). **E** Fluorescent intensity of Zinc in mouse sperm stained by ZnAF-1 and analyzed by flow cytometry (*n* = 5). **F** Western blot detection of ZIP12 protein in CD (*n* = 3) and HFD (*n* = 8) mice. The relative levels of ZIP12 protein were normalized to β-actin. Data are shown as means ± SEM. *P < 0.05, **P < 0.01, ****P < 0.0001

In general, a common characteristic of obesity is that there is a similarity between the status of systemic and tissue-local oxidative stress. To probe into the role of ZIP12 and zinc influx in protecting sperm against oxidative stress, we determined if there is a correlation between obesity related oxidative stress and ZIP12 expression levels in vivo. To make this assessment, male mice were fed a high-fat diet (HFD) for 10 weeks to ensure that they gained significantly more body weight compared to their age-matched littermates fed instead a normal control diet (CD) (Fig. 4A). These obese males (HFD group) exhibited significantly decreased sperm motility and increased incidence of sperm abnormality in comparison to that in the CD group (percentage of motility decline: 38.39 ± 3.46 vs. 77.96 ± 2.38, *n* = 10, *P* < 0.05; sperm abnormality rate: 66.65 ± 4.40 vs. 44.09 ± 5.78, *n* = 10, *P* < 0.05) (Fig. 4B, C). Such poor sperm quality in obese mice (HFD group) was associated with a significantly higher level of ROS generation (Fig. 4D) and lower zinc content (Fig. 4E) detected by flow cytometry analysis. These differences suggest that there is an association between declines in zinc content which can cause ROS generation to rise to levels that induce oxidative damage in sperm.

On the other hand, despite the increases in oxidative stress in the HFD group, Western blot analysis showed that the testicular ZIP12 protein expression declined relative to that in the CD mice testes (Fig. 4F). Thus, this inverse correlation between declines in ZIP12 expression levels and rises in ROS generation in HFD fed mice suggested that ZIP12-induced dynamic transport of zinc into spermatogenic cells or spermatozoa may be crucial for preventing sperm cells from oxidative damage during spermatogenesis.

### H_2_O_2_ / sodium palmitate-induced oxidative stress downregulates ZIP12 expression in C18–4 cells

**Fig. 5:**
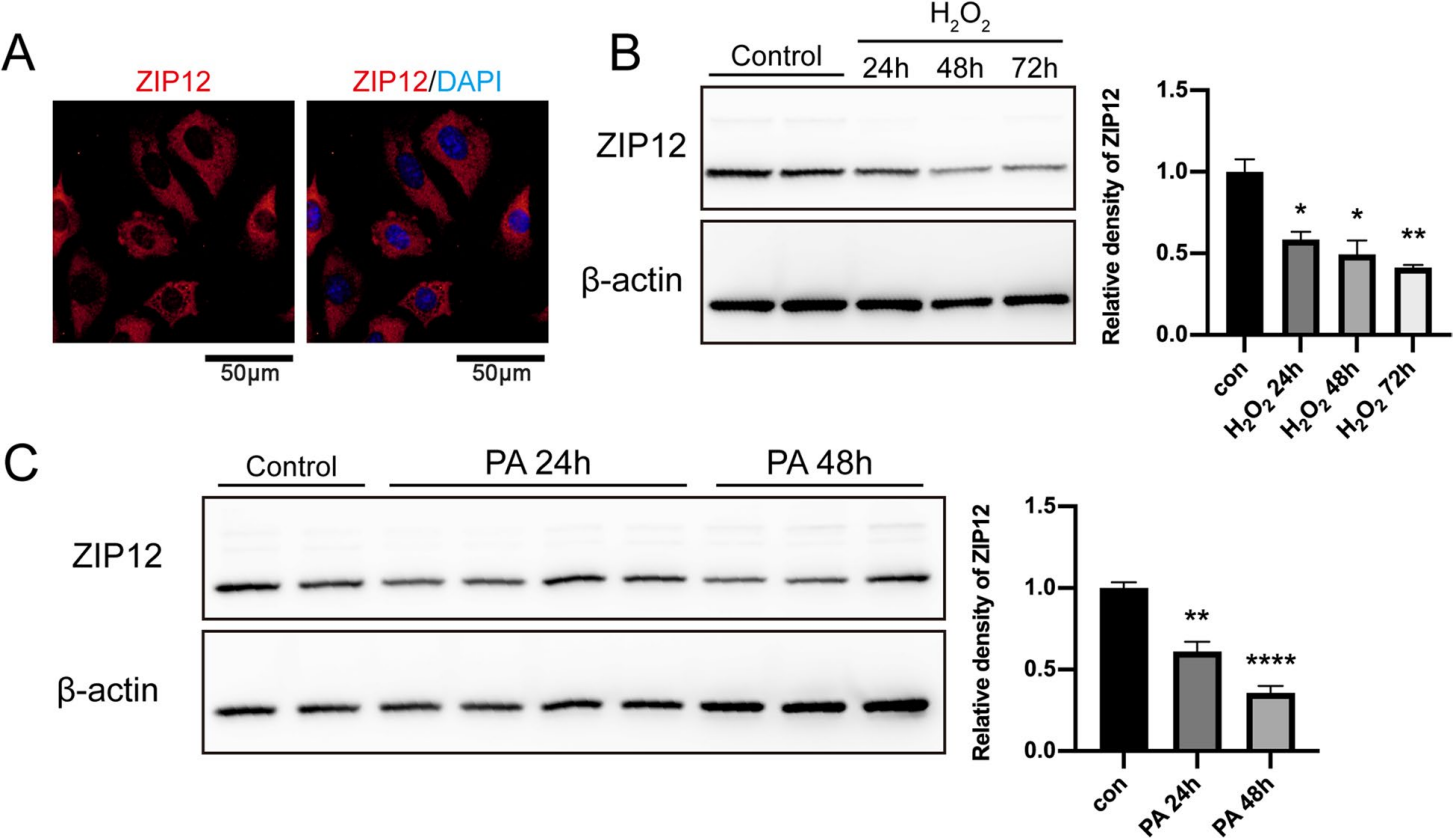
ZIP12 expression in response to oxidative stress in the C18–4 spermatogonia cell line. **A** Immunofluorescent staining of ZIP12 (red) in C18–4 cells. Cell nuclei were stained with DAPI (blue). Scale bar = 50 μm. **B** Western blot detection of ZIP12 protein expression levels in C18–4 cells after 100 μM H_2_O_2_ treatment for 24 h, 48 h, 72 h. The relative levels of ZIP12 protein were normalized to β-actin. Data are shown presented as means ± SEM from three independent experiments. **C** Western blot detection of ZIP12 protein expression levels in C18–4 cells after 50 μM PA treatment for 24 h and 48 h. The relative levels of ZIP12 protein were normalized to β-actin. Data are presented as means ± SEM from six independent experiments. *P < 0.05, **P < 0.01, ****P < 0.0001

Since the expression of ZIP12 was prominent in the spermatogonia in the seminiferous epithelium, a mouse spermatogonia cell line, the effects were evaluated of losses in ZIP12 expression on oxidative stress induced damage in C18–4 cells. Both immunofluorescence staining and Western blot analysis confirmed the expression of ZIP12 in C18–4 cells (Fig. 5A-C). After exposure to a ROS inducer H_2_O_2_ (100 μM) for 24 h, 48 h, and 72 h, ZIP12 expression levels in C18–4 cells significantly decreased (Fig. 5B). Similarly, ZIP12 expression also declined in C18–4 cells when exposed to a lipotoxic condition induced by sodium palmitate (PA) treatment (200 μM), which also promotes ROS generation (Fig. 5C). The decreased expression of ZIP12 in C18–4 cells under oxidative stress was consistent with that in testes from obese mice fed a HFD, which suggested that oxidative stress can downregulate ZIP12 expression in testes or spermatogonia, while inadequate amounts of ZIP12 in spermatogonia potentially contributes to insufficient antioxidant defense against oxidative damage.

### ZIP12 knockdown reduces intracellular zinc content and antioxidant capacity in C18–4 cells

**Fig. 6:**
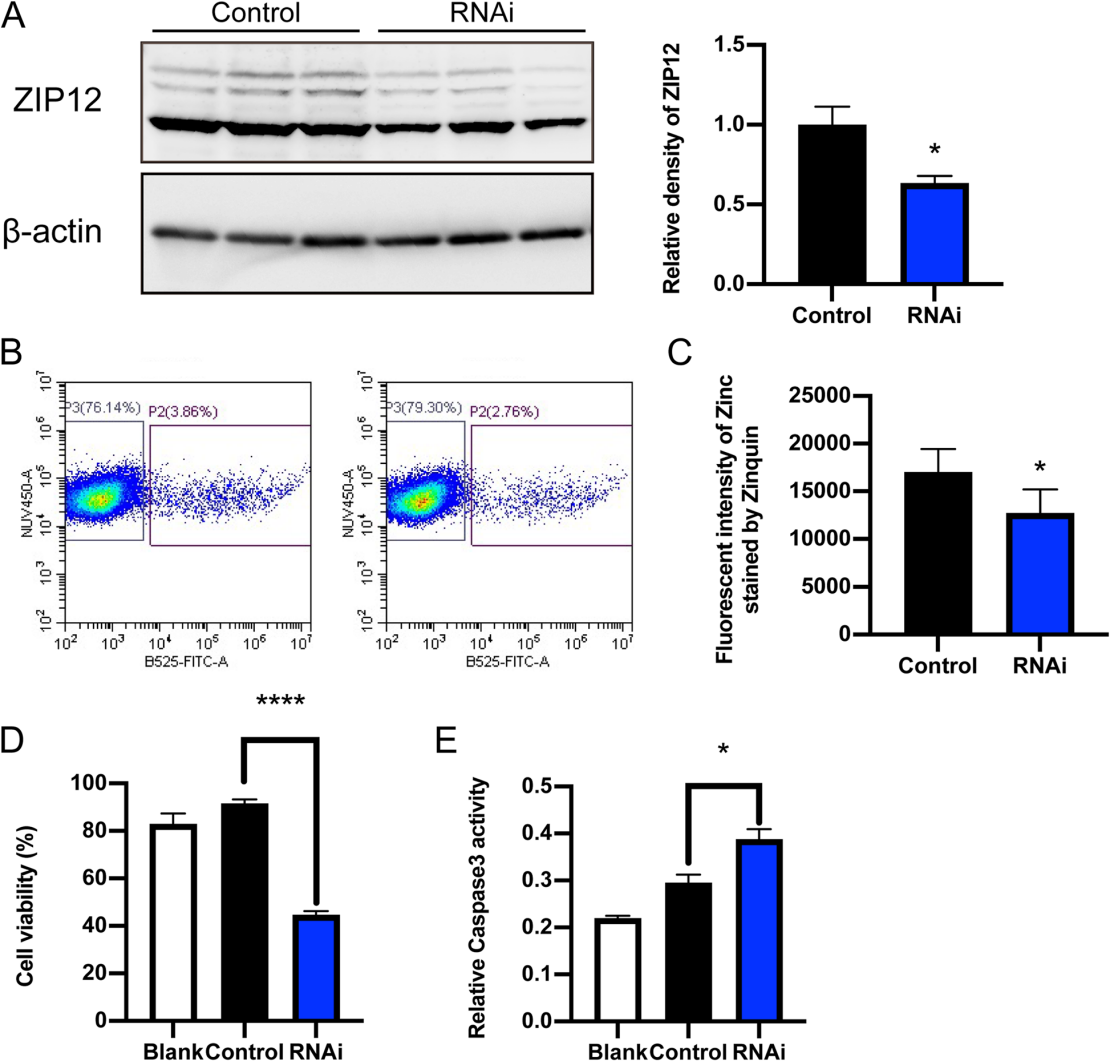
Knockdown of *Zip12* decreased zinc content and the antioxidant capacity of C18–4 cells. **A** Western blot analysis of ZIP12 protein expression levels in the control and the ZIP12 shRNA transfected group. The relative levels of ZIP12 protein were normalized to β-actin. Data are shown presented as means ± SEM from three independent experiments. **B-C** Flow cytometric analysis of the fluorescence intensity of zinc on the control (*n* = 5) and the *ZIP12* shRNA transfected group (*n* = 5). **B** One representative sample showed P2 region, which represented cells transfected with control shRNA or *ZIP12*-specific shRNA that were positive for GFP signal. **C** Mean fluorescence intensity of zinc of the two groups indicated a decrease in the zinc level in *ZIP12*-knock down C18–4 cells. Data were presented as means ± SEM from four independent experiments. **D** CCK-8 proliferation assay in control shRNA (*n* = 4) or ZIP12-specific shRNA (*n* = 4) transfected C18–4 cells with the treatment of 100 μM H_2_O_2_. Data are shown as means ± SEM from four independent experiments. **E** Relative Caspase-3 activity in C18–4 cells transfected with control shRNA or *ZIP12*-specific shRNA and treated with 100 μM H_2_O_2_. Data are shown as means ± SEM from three independent experiments. *P < 0.05, ****P < 0.0001

Loss of ZIP12 gene function was obtained through transfecting C18–4 cells with physiologically relevant *Zip12* siRNA to evaluate the antioxidant capacity of ZIP12 in spermatogonia. The expression of ZIP12 protein in C18–4 cells transfected with *Zip12*-specific shRNA significantly decreased compared to that in control group transfected with control shRNA (Fig. 6A). Then, the intracellular zinc stained by Zinquin, a fluorescent probe, was analyzed by flow cytometry. Consistent with the previous report in neuro-2a cells [32], the mean fluorescent intensity of intracellular zinc significantly decreased following *Zip12* shRNA knockdown in C18–4 cells (positive for GFP signal) (Fig. 6B, C), suggesting that ZIP12 was a key player responsible for maintaining intracellular zinc concentration.

Furthermore, H_2_O_2_ treatment was applied to assess if the antioxidant capacity of ZIP12. C18–4 cells in the control group and the *Zip12*-shRNA group were treated with H_2_O_2,_ respectively, and their proliferative and apoptotic activities were evaluated based on the results of the CCK8 assay and measurements of Caspase-3 activity. In the *Zip12*-shRNA group, its CCK8 level was lower whereas the Caspase-3 activity was higher than those in the control group (Fig. 6D, E), which indicated lower cell viability and higher apoptosis activity in *Zip12* knockdown cells. Taken together, insufficient ZIP12 expression levels accompanied with decreased intracellular zinc content in C18–4 cells can indeed impair the cell antioxidant capacity and finally result in higher sensitivity to oxidative stress and damage to spermatogonia and sperm homeostasis.

## Discussion

Zinc is an essential element with a wide range of biological functions and plays a significant role in the male reproductive system. However, the zinc transporters responsible for transmembrane zinc traffic during spermatogenesis are still not well understood. In the present study, we firstly clarified the expression of zinc transporters in testis and identified a key zinc transporter ZIP12 in spermatogonia. We demonstrated abundant expression of ZIP12 in testes, especially in spermatogonia and spermatozoa of both mice and humans, and then confirmed its crucial role in maintaining intracellular zinc content and its essential antioxidant ability in reducing losses in functional spermatogonia and sperm activity as well as fertility. Importantly, exposure to imposed oxidative stress reduced the expression of ZIP12 in testes or spermatogonia and thereby their zinc content fell. Therefore, our study strongly suggested that ZIP12 plays an indispensable role in the maintenance of intracellular zinc uptake at levels that relieve impairment of spermatogenesis and blunting losses in sperm quality. These deleterious effects can be encountered if declines in Zn^2+^ content in sperm and spermatogonia resulting from losses in ZIP12 function can no longer blunt the environmental stress-induced rises in ROS generation that can be injurious to spermatogonia and sperm functional integrity and fertility.

Zinc is a major non-enzymatic antioxidant in sperm and the male genital tract. Numerous studies proved the protective effect of zinc on male fertility and several clinical studies demonstrated the beneficial effects of zinc addition in vitro on sperm quality and the fertilization rate in assisted reproductive techniques [35–37]. In order to clarify the mechanisms underlying these therapeutic effects of zinc supplementation on spermatogenesis, we show here that adequate ZIP12 function is essential to maintain the intracellular testicular zinc sperm and spermatogonia content at levels that are sufficient to blunt stress-induced rises in ROS generation that can be injurious to their function and fertility. Even though we only have obtained a partial description of all the events that underlie the protective effects of zinc supplementation, we hope that our results will ultimately lead to improved therapeutic management of male infertility.

It is noteworthy that a recent clinical study including 2370 couples investigated the effect of dietary supplements containing zinc and showed that zinc supplementation does not appear to improve pregnancy rates, sperm counts or sperm function [38]. A possible reason for this negative result is that the dietary zinc uptake into the circulation is different to that in the testicular tissues [39]. Another possibility is that zinc content was adequate and not limiting in the testicular microenvironment. Therefore, the spermatogenic cells were mainly dependent on zinc traffic afforded solely by zinc transporters including ZnTs and ZIPs. These two classes of zinc transporters are well-documented to mediate the transfer of zinc across physiological membranes and maintain the proper intracellular zinc concentration.

Previous studies have preliminarily revealed their physiological functions of ZnTs and ZIPs mainly based on their distribution. For instance, ZnT1 can regulate zinc absorption and secretion depending on its specific location at the apical or basolateral membrane. ZnT4, ZnT5 and ZnT6 are distributed in the inner membrane of organelles and responsible for zinc transport into organelles [40]. ZIP4 is mainly expressed in human epidermal keratinocytes, and its deficiency leads to acrodermatitis enteropathica [41]. On the other hand, ZIP5, ZIP6, and ZIP10 are expressed in the testis [34]. Specifically, ZIP5 is expressed in the plasma membrane of Sertoli cells, while ZIP6 and ZIP10 are mainly distributed in the plasma membrane of spermatozoa [34], which suggests that they have roles in controlling the zinc influx in these cells. However, the expression and function of zinc transporters in the male germ cells were rarely reported and their roles in zinc homeostasis remain unclear during exposure to an oxidative stress induced by pathological conditions or environmental challenges.

There is an increasing awareness that a trend zinc content increase during different periods of spermatogenic cell development. During spermatogenesis sufficient intracellular zinc is crucial for maintaining cell function [1, 12]. Accordingly, we focused on the expression of ZIPs in testis owing to their functions in mediating zinc influx into cells. In the present study, we analyzed the expressions of all 14 ZIP members in mouse testis and found significantly high expression of ZIP12 in testis in comparison to other ZIPs. Moreover, although ZIP12 is ubiquitously distributed in tissues, its mRNA and protein levels were at higher levels in the testis than in other body tissues. Considering the high testicular zinc content, the relatively high testicular level of ZIP12 expression is consistent with the notion that it has a potentially indispensable role in zinc homeostasis and spermatogenic cell development.

Previous studies on ZIP12 were mainly focused on its function in the central nervous system [33, 42–44]. They showed its important role in neuronal differentiation. Meanwhile, ZIP12 was also reported to participate in hypoxia-induced remodeling of pulmonary arterioles [45]. It is noteworthy that ZIP12 has the highest affinity to zinc among all of the ZIP transport proteins [46] and the loss of ZIP12 is indispensable because it cannot be compensated for by other zinc transporters in the central nervous system [33]. However, the expression and function of ZIP12 has never been reported before in the testis or spermatozoa.

In the current study, we firstly found that ZIP12 was highly abundant in spermatogonia and spermatozoa, both in mice and humans. Spermatogonia are the primordial cells of spermatogenesis, and any damage to spermatogonia may result in a spermatogenic disorder and male infertility. Thus, the high level of ZIP12 expression in spermatogonia is suggestive of an important role in zinc homeostasis during spermatogenesis. Meanwhile, the corresponding agreement between the delimited high levels of ZIP12 expression and localized increased levels of intracellular zinc content in the spermatozoa are indisputable. This concordance confirms its role in mediating zinc influx which is potentially involved in blunting the environmental-induced rises in ROS generation that can induce oxidative damage that may impair the maintenance of sperm function and quality.

To explore the role of ZIP12 in defense of oxidative stress, an obese mouse model was employed to investigate the correlation between ZIP12 expression level and sperm quality. Obesity is a well demonstrated cause of systemic and tissue-local oxidative stress and thereby leads to declines in sperm quality [47, 48]. Previous studies reported that zinc contents in the skin, muscle, and bone is reduced in obese mice [49], and serum zinc concentration is significantly lower in obese children than that in matched normal-weight children [50–52]. Meanwhile, the expressions of some zinc transporters, such as ZNT1 and ZNT6 in brain [53], ZIP14 in adipose [54, 55], and ZnT4, ZnT5, ZnT9, ZIP1, ZIP4 and ZIP6 in leukocyte [56], were inversely correlated with obesity. Herein, our study provided similar evidence that ZIP12 expression in testis significantly decreased in obese mice, which is associated with reduced sperm zinc content, high sperm ROS level, poor sperm quality and male subfertility. Thus, reduced ZIP12 expression in testis may be a cause of insufficient zinc content and elevated oxidative stress in sperm, which may further lead to male infertility.

Considering the role of ZIP12 in supporting zinc homeostasis or antioxidant defense, we were curious in clarifying how obesity downregulates testicular ZIP12 expression. A previous study showed that oxidative stress reduces expressions of zinc transporters in hepatocytes [57]. Due to the tight association between obesity and oxidative stress, our results also demonstrated a decrease of ZIP12 expression in response to oxidative stress in a spermatogonia cell line C18–4 cells. Consistent with its reduced expression in obese testis, ZIP12 expression in C18–4 cells significantly declined in response to the H_2_O_2_ or PA induced oxidative stress. Therefore, our study documented that downregulated ZIP12 expression induced by oxidative stress can subsequently lead to a decrease of zinc-associated antioxidant activity, and thereby aggravate oxidative stress in turn.

Furthermore, the role of ZIP12 functional expression in supporting spermatogonia homeostasis was also evaluated through *Zip12* knockdown in C18–4 cells. Consistent with the previous report in neuro-2a cells [32], we also found that *Zip12*-shRNA knockdown decreased intracellular zinc content in C18–4 cells. In addition, our results indicated that ZIP12 played a crucial role in defending against oxidative damage and the maintenance of cell viability. This is evident since reduced ZIP12 expression in C18–4 cells resulted in insufficient antioxidant capacity and rendered the cells prone to undergo apoptosis. It should be pointed out that, we are aware of the limitation of RNAi assay in vitro in the current study. Thus, additional studies in vivo, such as *ZIP12* knock-out mice, are required to understand the precise role of ZIP12 in the spermatogonia involved in spermatogenesis and male fertility.

## Conclusions

**Fig. 7:**
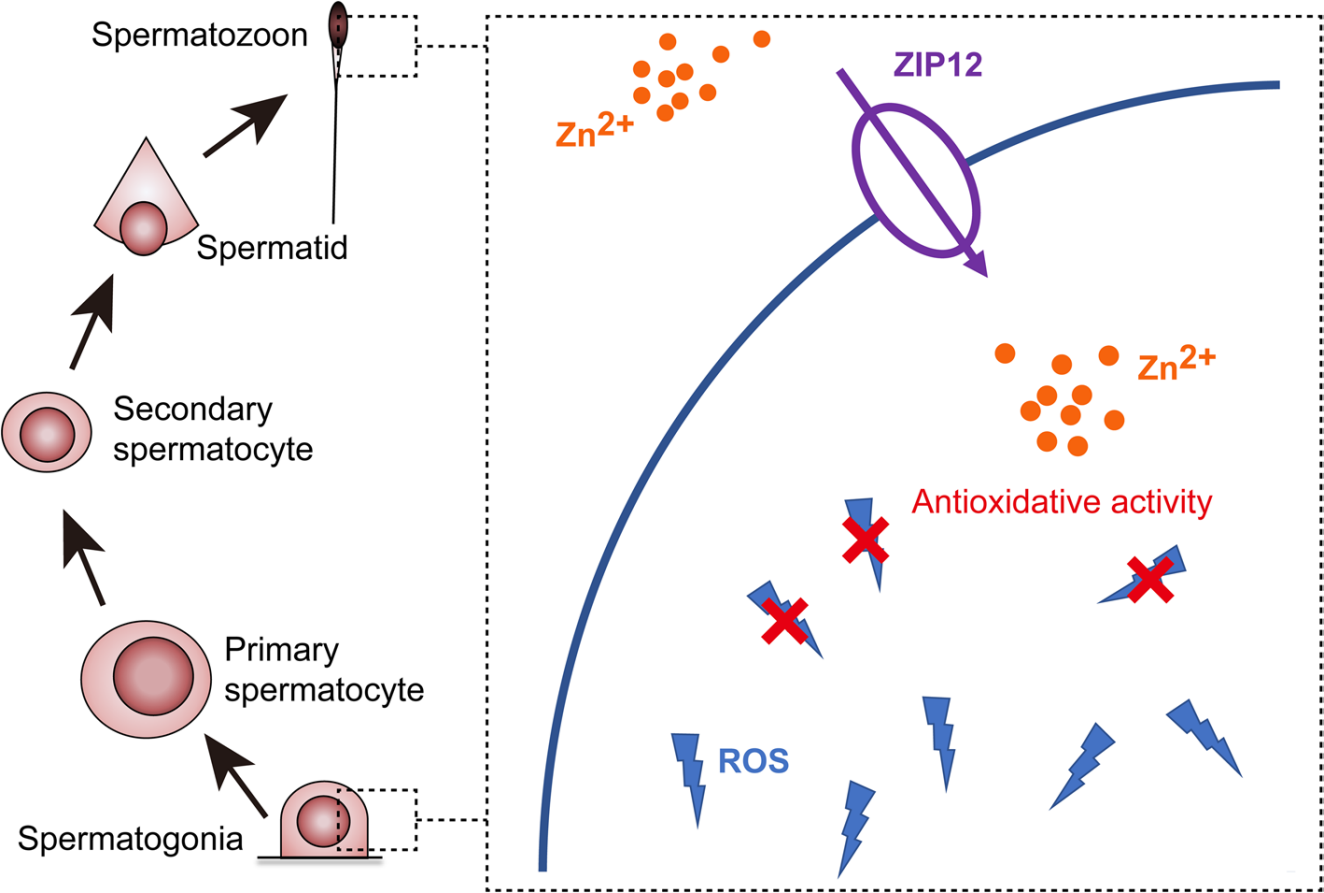
Schematic representation of ZIP12 function in spermatogenesis. The zinc transporter ZIP12 is highly expressed in the testis, especially in spermatogonia and spermatozoa. It mediates zinc influx into cytosol and is involved in maintaining intracellular zinc content at levels that are adequate to play a key role in suppressing environmental stress-induced rises in ROS from reaching levels that may be injurious to spermatogonia and sperm quality

In summary, a zinc transporter ZIP12 is highly expressed in the testis, especially in spermatogonia and spermatozoa. It participates in mediating zinc influx and is crucial for zinc homeostasis during spermatogenesis. By maintaining intracellular zinc at high enough levels, ZIP12 may play a key role in reducing oxidative stress in spermatogonia and spermatozoa (Fig. 7). In contrast, insufficient ZIP12 accompanied with decline in intracellular zinc level can impair antioxidative capacity and finally lead to male subfertility or infertility.

## Supplementary Information

**Additional file 1: Supplementary Table 1.** PCR primers.

## Data Availability

All data generated or analyzed during this study are included in this published article and its supplementary information files.

## Abbreviations

CASA: Computer-assisted sperm analysis
CCK-8: Cholecystokinin-8
CD: Control diet
DAPI: 4′,6-diamidino-2-phenylindole
DIC: Differential interference contrast
GFP: Green fluorescent protein
H_2_O_2_: Hydrogen peroxide
HFD: High-fat diet
IF: Immunofluorescence
IHC: Immunohistochemistry
PA: Sodium palmitate
PLZF: Promyelocytic leukemia zinc finger
RNAi: RNA interference
RT-qPCR: Quantitative reverse transcription polymerase chain reaction
SE: Standard Error
SEM: Standard error of mean
shRNA: Short hairpin RNA
SOD: Superoxide dismutase
SPSS: Statistic package for social science
ZIP: Zrt-, Irt-like protein
ZnT: Zinc transporter protein
